# Catalytic activity of ethylbenzene with product selectivity by gold nanoparticles supported on zinc oxide

**DOI:** 10.55730/1300-0527.3363

**Published:** 2021-12-28

**Authors:** Afiq ANWAR, Azman MA’AMOR, H.N.M. Ekramul MAHMUD, Wan Jefrey BASIRUN, Iskandar ABDULLAH

**Affiliations:** Department of Chemistry, Faculty of Science, University Malaya, Kuala Lumpur, Malaysia

**Keywords:** Gold nanoparticle, ethylbenzene, oxidation reaction, gold catalysis, characterization

## Abstract

The oxidation of ethylbenzene (EB) using *tert*-butyl hydroperoxide as the oxidizing agent was carried out in presence of gold nanoparticles (3 nm) supported on zinc oxide in acetonitrile solution. A higher selectivity towards acetophenone (ACP) as the major product, and a moderate selectivity towards other products such as 1-phenylethanol (PE), benzaldehyde (BZL), and benzoic acid (BzA) were observed using the prepared Au/ZnO nanocatalysts at 100 °C for 24 h. It is suggested the reaction produces an intermediate product, which is dimethylethyl^−1^-phenylethyl peroxide through a radical mechanism. A small amount of benzaldehyde was observed because benzaldehyde went autoxidation to form benzoic acid with the presence of oxidation agent of TBHP during reaction. The factors affecting the catalytic activity such as gold loading, calcination treatment at 300°C, type of solvent, reaction time, reaction temperature, oxidant to substrate molar ratio, catalyst weight, and solvent volume were studied. The gold nanoparticle catalyst synthesized by deposition precipitation method using urea was characterized by XRD, HRTEM, ATR-IR, XRF, and BET and offers a very selective reaction pathway for the oxidation of ethylbenzene.

## 1. Introduction

Large particle size of gold appears to be a poor catalytic material compared to palladium, platinum, or ruthenium. Nanoscale gold catalyst is widely regarded as the best green catalytic material for various processes and applications such as selective oxidation of alcohols [ 1], hydrogenation of nitro compounds [2], laser ignition [3], cosmetic [4], photocatalysis [5,6 ], and glycosylation of carbohydrate [ 7]. Catalytic Au nanoparticles (NPs) have great prospects in environmentally friendly and green sustainable chemical processes because of their high activity at room temperature and possessing high oxygen storage capacity, which is suitable for oxidation reaction [8]. Zinc oxide (ZnO) was used as a support material, as this metal has been widely applied in various organic reactions, especially in photocatalytic [9] and oxidation reactions [10,11 ]. ZnO is a cheap material with excellent surface properties, which increases the catalytic activity. The preparation of gold on ZnO support via the deposition-precipitation method produced gold NPs with a narrow particle size distribution of less than 5 nm [ 12]. The main products reported for the oxidation of ethylbenzene are acetophenone, 1-phenylethanol and benzaldehyde, and the precursors in pharmaceutical products such as hydrogels, hydrazones, and benzalacetophenones (chalcones) [13]. The catalytic oxidation of ethylbenzene to acetophenone (ACP) has drawn much attention for the past few years, as it is used as a fragrance in soaps, perfumes and cosmetics; as a flavouring agent in foods; as a solvent for plastics and resins; and in the synthesis of pharmaceutical drugs [14]. At present, the industrial production involves high temperature thermal autoxidation in the absence of a heterogeneous catalyst. The oxidation process requires potassium permanganate and potassium dichromate, which produce toxic, dangerous, and hazardous by-products [15]. Oxidants such as *tert*-butyl hydroperoxide (TBHP), hydrogen peroxide, N-hydroxyphthalimide, iodoxybenzoic acid (IBX), and oxygen could successfully convert alkyl benzene into the desired products [16–18]. It is well known that gold supported metal oxides are very active oxidants for CO oxidation [19]. However, no effort has been made for the oxidation of ethylbenzene on Au NPs supported on ZnO. Thus, the main challenge is to achieve a higher conversion of ethylbenzene oxidation, since it is difficult to activate the ethyl carbon for the oxidation reaction. Most of the ethylbenzene oxidation with heterogeneous catalysts demonstrates a lower conversion [13,20, 21 ]. Other catalysts such as zeolite Beta containing ultra-small CoO particles and MnFeSi composite also demonstrated low conversions for the oxidation of ethylbenzene, which produce acetophenone as the major product, together with 1-phenylethanol, 1-phenylethyl hydroperoxide, benzoic acid and benzaldehyde as the by-products [22, 23]. It is imperative to develop a green or environmentally friendly nontoxic catalytic system with good stability, higher conversion, and selectivity. Hence, the focus of this research is to synthesise and optimize gold nanoparticles supported on zinc oxide catalysts for the oxidation of alkyl benzene, since gold catalyst has excellent selectivity towards the target product [24–26]. The synergistic effects of using Au nanoparticles with ZnO can be realized from this work to obtain higher conversion of ethylbenzene.

## 2. Materials and methods

All chemicals were of analytical grade with high purity. Acetonitrile for analysis (99.5 %) and anisole (99%) were from Emsure Merck, urea (AR grade) from Friendemann Schmidt, phenylethyl alcohol from Acros, gold (III) chloride trihydrate and zinc oxide nanopowder from Sigma–Aldrich. While other analytical standards such as benzaldehyde (99%), ethylbenzene (99%), acetophenone (99%), benzoic acid (99%) and TBHP solution 70 wt% in water, were from Sigma–Aldrich.

### 2.1. Synthesis of gold nanoparticles supported on zinc oxide

The catalyst was prepared by deposition precipitation using urea, according to the method of Geus et al. [27]. ZnO nanopowder (< 50 nm) was used as the support material for gold loading. Urea (CO(NH_2_)_2_) was used as the precipitating agent that allowed the gradual and homogeneous introduction of hydroxide ion in the process, while preventing the local increase in pH value and the precipitation of metal hydroxide. ZnO nanopowder (1 gram) was predried at 100 °C for 24 h prior to the addition of 100 mL aqueous solution of the gold precursor, where the amount of gold corresponds to the maximum gold loading on ZnO. The weight % of gold in 1 g of ZnO were 0.2537 mM (0.5 wt%), 1.015 mM (2 wt%), 2.031 mM (4 wt%) and 4.06 mM (8 wt%). The deposition of gold onto ZnO was prepared in the excess of urea, with a ratio Au:urea of 1:100. The pH value of the mixture was 7 in the excess of urea. The mixture was then separated by centrifugation at 6000 rpm for 15 min at room temperature. The supernatant was removed, while the precipitate was dried at 100 °C for 2 h under vacuum, prior to calcination at 300 °C for 4 h at a rate of 2 °C min^−1^. The catalyst was stored in the dark at –5 °C.

### 2.2. Catalyst characterization

Shimadzu X-ray Fluorescence Spectrophotometer μ-EDX 1400 was used to analyse the metal loading in the catalyst. TriStar II 3020 Micromeritics nitrogen thermal adsorption instrument was used to calculate the total surface area, pore volume and sizes from the Brunauner-Emmet-Teller (BET) method. The average particle size and particle distribution were estimated using the Image J software analysis of the micrographs from a high-resolution transmission electron microscopy (HRTEM, JEOL JEM 2100-F), operated at 200 kV using formvar carbon film on a 400-mesh copper grid. The lattice parameters and degree of crystallinity were determined using X-ray diffraction (XRD, PANalytical model EMPYREAN) at ambient temperature with 2-theta scan range from 20° to 90°, with a Cu Kα radiation at 40 kV and 25 mA. The solvent remnants were analysed using Perkin–Elmer Spectrum 400 ATR–FTIR (attenuated total reflectance-Fourier transform infrared).

### 2.3. Catalytic oxidation experiment

Ethylbenzene (EB) was refluxed with anisole internal standard in acetonitrile solvent using 50 mg of synthesized Au/ZnO catalyst, 1:1 molar ratio of TBHP for 24 h at 100 °C with constant stirring. The reaction product was centrifuged at 6000 rpm for 10 min. The clean and filtered supernatant was collected and analyzed using an agilent gas chromatography flame ionization detector (GC-FID) with HP-5 semipolar column (composition 5% Phenyl 95% dimethylpolysiloxane). The inlet was set to a split mode with 50:1 ratio at 250 °C. Hydrogen was used as the carrier gas with flow rate flowrate at constant linear velocity at 40 cms^−1^.

## 3. Results

### 3.1. Characterization of catalyst

**Figure 1:**
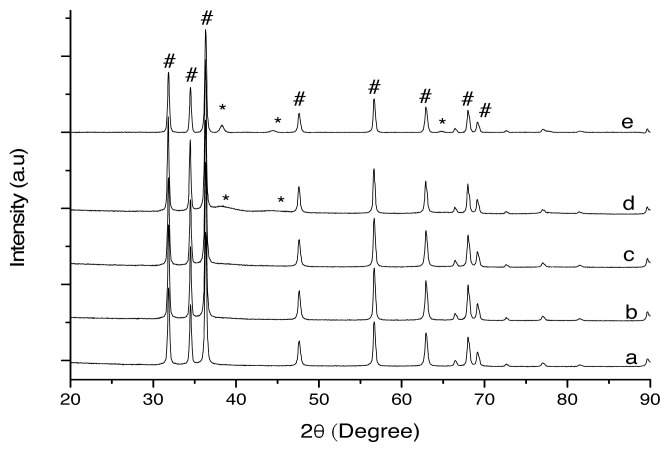
XRD diffractogram for (a) support material ZnO and various gold loading on zinc oxide, (b) 2.0 wt%, (c) 4.0 wt%, (d) 8.0 wt%, and (e) spent 4.0 wt%.

The XRD diffractograms of ZnO (a), 2.0 wt% Au/ZnO (b), 4.0 wt% Au/ZnO (c), 8.0 wt% Au/ZnO (d), and spent 4 wt% Au/ZnO (e) are shown in Figure 1. The diffractograms were compared with the standard database of the International Centre for Diffraction Data (ICDD). Figure 1 shows ZnO peaks (#) at 2θ = 32°, 34°, 36°, 47°, 56°, 63°, 66°, 68°, 69°, 72°, 77°, 81°, and 90° attributed to the (100), (002), (101), (102), (110), (103), (200), (112), (201), (004), (202), (104), and (203) planes respectively, which are similar to the results obtained in other studies [ 12,28, 29 ]. Figure 1(b) and Figure 1(c) show no Au peak detected as the Au is present in nanoparticle size. For crystallite sizes below 100 nm, line broadening occurs due to incomplete destructive interference in scattering directions where the x-rays are out of phase [ 30]. Ndolomingo et al. stated that XRD could not detect the presence of Cu and Au in the catalyst, as it is below the detection limit of 5 wt% of elemental Au [31]. The presence of gold with higher gold loadings could be detected at 38° as shown in Figure 1(d), where the small slope at this theta degree increases for loadings greater than 8.0 wt% Au/ZnO. Au peaks (*) are observed in Figure 1(e) after the oxidation reaction with gold peaks were observed at 2θ = 38°, 44°, and 65° corresponding respectively to the Miller indices of (111), (200), and (220), possibly due to the agglomeration of gold particle. The nanoparticle size of Au will be discussed further in subsection of HRTEM below. The low presence of gold in lower gold loadings is due to the smaller size of the gold nanoparticle (< 5 nm), which was confirmed from the HRTEM analysis.

**Figure 2:**
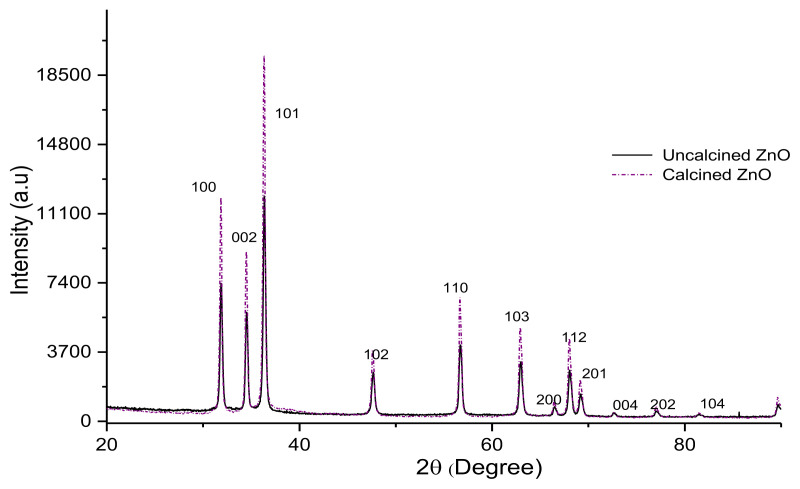
XRD diffractogram for uncalcined and calcined ZnO.

The ZnO diffraction peaks, on the other hand, had higher intensity after calcination compared to the uncalcined ZnO as shown in Figure 2, as the particles were more crystalline due to calcination [32]. The higher intensity in the XRD diffractograms corresponds to a higher degree of crystallinity with lower surface area [33]. This is the reason for the larger surface area of uncalcined ZnO (m^2^/g) compared to the calcined ZnO, as shown in Table 1.

**Figure 3:**
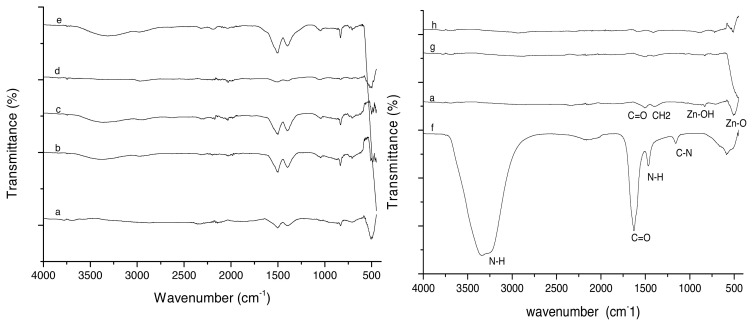
FTIR spectrum for (a) support material ZnO and various gold loading Au/ZnO, (b) 0.5 wt%, (c) 2.0 wt%, (d) 4.0 wt%, (e) 8.0 wt%, (f) urea sol, (g) uncalcined 4.0 wt%, and (h) spent 4.0 wt%.

Figure 3 shows the FTIR spectrum for (a) support material ZnO and various gold loading Au/ZnO, (b) 0.5 wt%, (c) 2.0 wt%, (d) 4.0 wt%, (e) 8.0 wt%, (f) urea sol, (g) uncalcined 4.0 wt%, and (h) spent 4.0 wt%. ZnO peaks are identified at 830 cm^−1^ (Zn-OH), 505 cm^−1^ (Zn-O), 1500 cm^−1^ (C=O) and a double peak at 1395 cm^−1^ (CH_2_) [34, 35 ], which are typical of the different amounts of Au/ZnO i.e. 0.5 wt%, 2.0 wt%, and 8.0 wt% with similar transmittance intensities. However, the (d) calcined Au/ZnO 4 wt%, (g) uncalcined 4 wt% Au/ZnO, and (h) spent 4 wt% of Au/ZnO show smaller intensities because these catalysts were freshly analysed, while the others were stored for 24 h in a refrigerator (5 °C). The FTIR spectrum also shows clean peaks for the uncalcined 4 wt% Au/ZnO catalyst (h) and spent 4 wt% Au/ZnO (after the oxidation reaction). It can be concluded that the catalysts were free from traces of urea with the absence of N-H peak (~3300 cm^−1^) after the synthesis [ 36].

The elemental compositions (wt%) of the catalyst were analysed using XRF and shown in Table 1. The prepared catalyst has slightly lower elemental percentage (Table 1, entry 4) compared to the expected weight percent (wt%). Whereas, the minimum gold loading is above the isoelectric point (IEP) of ZnO (pH 9.78) [12]. However, Zanella et al. [37] and Haruta et al. [38] reported that larger particle sizes could be produced when the pH is less than 7. The smaller presence of Au at low pH value is due to the hydrolysis of AuCl_4_^−^ precursor, which makes it difficult for the deposition of gold NPs on zinc oxide. When the solution’s pH value is higher than the IEP of metal oxides, the AuCl_4_^−^ precursor could not interact with the metal oxide surface due to the surface charge repulsion. This results in the leaching of the unreacted gold into the solution, thus, producing lower gold loadings [39–41]. Nevertheless, the dropwise addition of urea gradually increases the pH of the gold precursor (~pH = 2) to pH = 7 for increased gold loadings. Whereas, the sudden increase of high concentration of urea with the gold salt could lead to the fulmination of gold [42]. The presence of light could decrease the Au wt%, as light illumination is known to decompose the gold precursor [39]. The highest gold loading on ZnO was achieved at 7.1 wt% (Table 1, entry 8) following the urea approach in deposition precipitation method [40]. This higher loading by urea approach was due to the slow precipitation of hydroxide on the support allowing most of the gold to be deposited on the support which cannot be obtained by NaOH deposition method [12, 40, 43, 44 ].

The BET result (Table 1) shows the variance in the surface area, pore size and pore volume of various gold loadings on ZnO. It was observed that the deposition of gold on ZnO support increases the total surface area (m^2^/g), while the pore size slightly decreases as gold was deposited in the pores. Alabbad et al. also found that gold and silver had higher surface area when deposited on metal oxide [45]. Supposedly, the presence of gold on ZnO support has smaller surface area, as it is immobilized in the pores of the material compared to the pristine ZnO support [46]. This is also the reason for the lower gold peak (2θ = 38°) in the XRD diffractogram. Since the difference is fairly small, the value is within the measurement error which is ± 5.0 cm^2^ g^−1^ and ± 0.005 cm^2^ g^−1^ for the surface area and pore size, respectively. It is assumed that the size of Au NPs, which cover the surface area and fill the pore volume, is very small. However, the pore size shows a significant decrease due to the voids in ZnO mesopores occupied by Au NPs. This suggests an enhancement of the texture properties of mesoporous ZnO after Au deposition. This can be ascribed to the formation of smaller sized Au NPs, which increases the surface area and pore volume [35].

**Figure 4:**
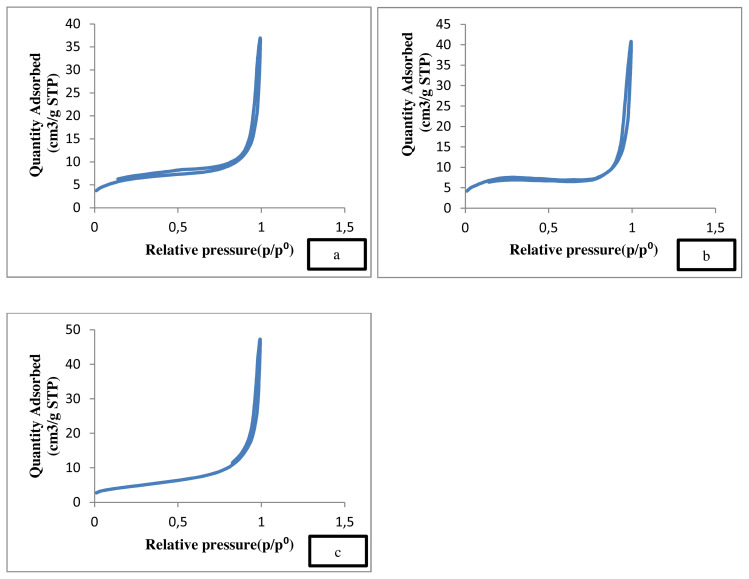
Type IV adsorption isotherm of (a) ZnO, (b) uncalcined 4 wt% Au/ZnO, and (c) calcined 4 wt% Au/ZnO.

Figure 4 shows the type IV adsorption isotherm. Apparently, the isotherm reveals a clear hysteresis loop when the relative pressure is in a certain range [ 47]. This illustrates a narrow pore size distribution of the ZnO supports and Au/ZnO catalyst.

**Figure 5:**
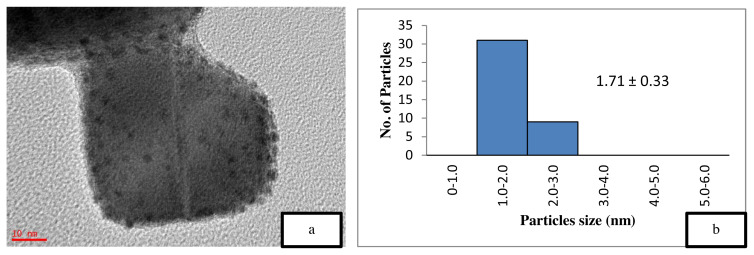
TEM image of (a) uncalcined 4 wt% Au/ZnO at 10 nm and (b) particle distribution graph.

**Figure 6:**
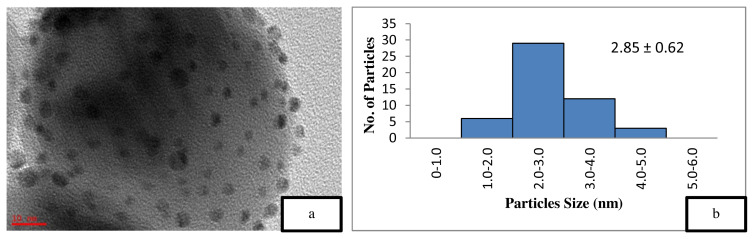
TEM image of (a) calcined 4 wt% Au/ZnO at 10 nm and (b) particle distribution graph.

**Figure 7:**
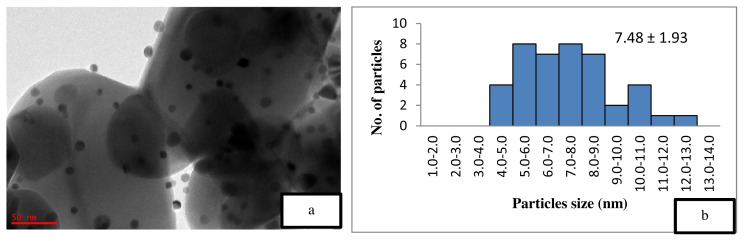
TEM image of (a) spent 4 wt% Au/ZnO at 50 nm and (b) particle distribution graph.

**Figure 8:**
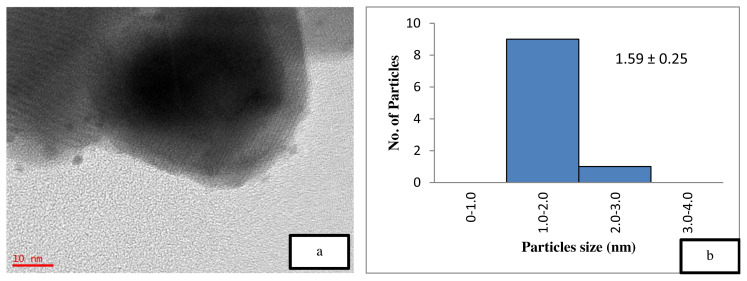
TEM image of (a) calcined 0.5 wt% Au/ZnO at 10 nm and (b) particle distribution graph.

**Figure 9:**
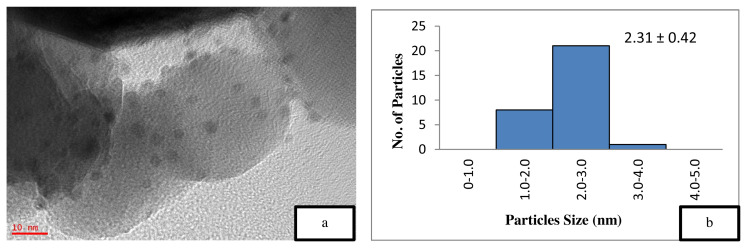
TEM image of (a) calcined 2.0 wt% Au/ZnO at 10 nm and (b) particle distribution graph.

**Figure 11:**
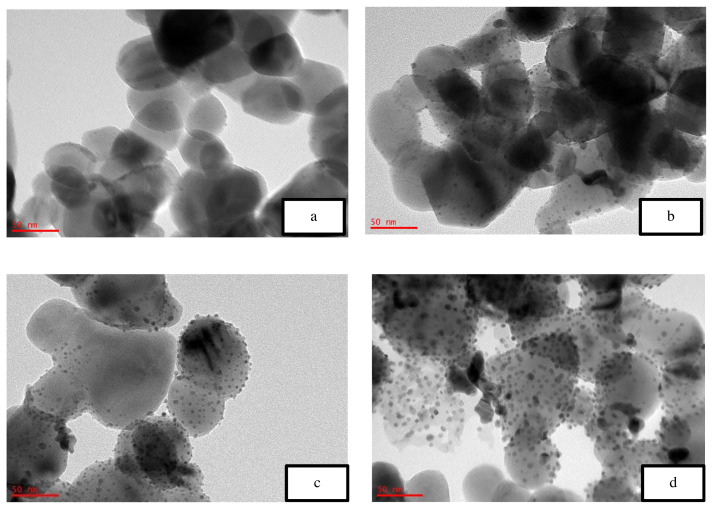
TEM image of calcined gold on zinc oxide for various gold loading at 50 nm (a) 0.5 wt%, (b) 2.0 wt%, (c) 4.0 wt%, and (d) 8.0 wt%

Figure 5 is the TEM image of the uncalcined 4 wt% Au/ZnO, which shows well distributed Au NPs on the ZnO, with an average particle size of 1.71 nm. The average particle size of Au NPs is 2.85 nm shown in Figure 6 after the calcination process. However, after the oxidation reaction, the average particle size increases to more than 5 nm and is randomly distributed as shown in Figure 7. The freshly synthesized Au/ZnO catalyst at different gold loadings shows well distributed Au NPs with an average particle size smaller than 3 nm, even after calcination, as shown in Figure 8, 9, and 10. As the gold loading is increased as shown in Figure 11, the void between the particles becomes smaller, hence has a higher tendency for faster agglomeration. This is in agreement with the study by Wang et al., where the size of Au NPs over the metal support is between 1 and 5 nm with a narrow size distribution [48]. The shape of the Au NPs on the surface of ZnO is almost hemispherical, indicating a strong metal-oxide interaction, which could enhance the catalytic activity [49]. The smaller size of the Au NPs in uncalcined, and calcined samples is the main reason for the absence of gold peaks in the XRD diffractogram. The Au peaks could only be detected by XRD when the size of Au is larger than 5.0 nm, as in the spent catalyst. The TEM images of all gold loadings in Figure 11 show uniform mesoporous structures (2–50 nm), and some of the images consist of large dark spots, since they are oriented parallel to the zone axis (Bragg contrast). Another factor is the overlapping of the powder catalyst at the back of the copper grid during the sample preparation in TEM analysis, which blocks the beam showing a darker contrast in the image. Nevertheless, the Au NPs is visible, and the sizes could be measured by ImageJ software.

### 3.2. Catalytic studies

**Figure 12:**
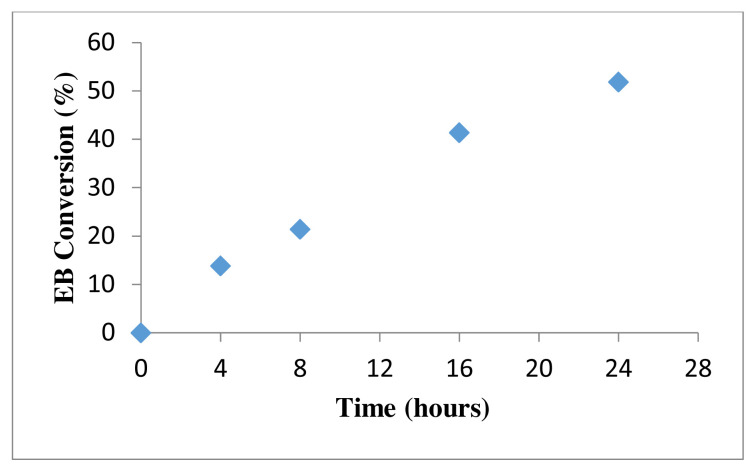
Catalytic activity of 0.2 M ethylbenzene oxidation using 50 mg of 4 wt% Au/ZnO.

The catalytic activity of EB oxidation using 50 mg of 4 wt% Au/ZnO at 100 °C with 0.2M EB and 1 mmol of TBHP as oxidation agent is shown in Figure 12. The conversion increased linearly from 0 h to 24 h reaction in which the conversion reaches 54.6 % conversion.

The products detected from the reaction were acetophenone (ACP), 1-phenylethanol (PE), benzaldehyde (BZL), and benzoic acid (BzA). A critical review was reported for the oxidation of alcohols and alkanes by employing supported gold nanoparticles as catalyst [50]. The proposed reaction mechanism for Au/ZnO catalysed ethylbenzene oxidation using TBHP as the oxidant under reflux is shown in Scheme 1 where TBHP activates the catalyst by providing oxygen atoms. Martins and coworkers discussed the oxidation of both benzyl alcohol and cyclohexane to benzaldehyde or acetophonenone and cyclohexanol or cyclohexanone, respectively by using supported gold nanoparticles on reusable catalysts with with H_2_O_2_ or TBHP [43]. The fact that initial controlled experiment in the absence of Au with H_2_O_2_ or TBHP gave no conversion proved the crucial role of the nanoparticles in catalysing the oxidation reaction efficiently. The reaction mechanism for the production of ACP, PE, BZL, and BzA from ethylbenzene as the starting material was thoroughy discussed by Tang and coworkers [51]. Following the free radical mechanism, the homolytic cleavage O-O bond of the TBHP produces *tert*-butoxy radical (t-BuO^•^) and hydroxy radical (HO^•^) species. Hydrogen abstraction from TBHP produces *tert*-butylperoxy radical (t-BuOO^•^) species when the kinetically favoured t-BuO^•^ abstracts the hydrogen as compared to the thermodynamically HO^•^ species.


$${\text{CO}}_{2}+{\text{H}}_{2}\text{O}={\text{H}}_{2}{\text{CO}}_{\text{3}}={\text{H}}^{+}+{{\text{HCO}}^{-}}_{3}$$

The former radical species, then, abstracts the benzylic hydrogen of EB generating 1-phenylethyl radical species and t-BuOH. The latter radical species, t-BuOO^•^, forms new C-O bond with the newly formed 1-phenylethyl radical species to form 1,1-dimethylethyl^−1^-phenylethyl peroxide, which subsequently undergoes decomposition *via* O-O homolytic cleavage to produce acetophenone and t-BuOH. 1,1-dimethylethyl^−1^-phenylethyl peroxide was not observed in this work, but the reaction is in agreement with William and coworkers that was reported on the thermal decomposition of 1,1-dimethylethyl^−1^-phenylethyl peroxide experiment [52]. 1-phenylexthoxyl radical also undergoes similar condition to produce benzaldehyde and further oxidized to benzoic acid.

### 3.3. Effect of calcination

The catalyst should be freshly calcined before reaction to prevent the decomposition and agglomeration of Au due to the presence of moisture and oxygen [39,45, 53 ]. A comparative study on the effect of calcination towards the catalytic activity of gold supported zinc oxide catalyst in ethylbenzene oxidation was performed, as illustrated in Table 2. The calcined 4 wt% Au/ZnO showed better catalytic activity compared to the uncalcined catalyst, which could be due to the increased crystallinity of ZnO [ 54]. This assumption is confirmed by the XRD data, which showed an increase in the crystallinity of the support after the calcination process. Jain et al. reported that the increase in crystallinity enhances the support reduction properties and the degree of metal-support interaction, thus, improving the catalytic activity [55]. On the other hand, Mingming claimed that the lower catalytic performance of the uncalcined catalyst is due to the presence of excess Cl precursor, which covers the Au surface during the synthesis process [53]. Thus, TEM was performed on the catalysts to study the effect of calcination on the Au/ZnO particle size. The smaller nanoparticle size is one of the main reason for the high performance of the catalytic material [13,37, 56 ]. However, the smaller Au NPs in 4 wt% uncalcined Au/ZnO (Table 2, entry 4) gave a lower conversion of ethylbenzene compared to the 4 wt% calcined Au/ZnO (Table 2, entry 5) with larger Au NPs size. This is due to the loosely held gold on the support material (ZnO) where the Au metal is not activated. According to Zanella et al. calcination is required to reduce Au^3+^ to Au^0^, even though thermal treatment could lead to larger particle sizes, provided that the particle growth is not drastic [37]. The reduction of Au^3+^ to Au^0^ could be observed from the colour change to dark purple, which is due to the plasmon resonance at ~550 nm [57]. Further analysis on the presence of reduced gold can be performed with the UV-Vis method. From the BET results in Table 1, it can be observed that the calcination decreases the surface area, thus, raising the demand for pore volume, pore size, and the degree of crystallinity. Figure 5 also shows the particle size distribution of the uncalcined and calcined catalyst. From the graph, the calcination resulted in the presence of larger particles of Au but did not significantly influence the total fraction of particles with sizes between 3 and 5 nm thus did not decrease the conversion of ethylbenzene. Thus, the calcination process plays an important role in the activation of gold nanoparticles by removing a significant amount of precursor residues on the catalyst, which exposes the Au NPs surface for enhanced catalytic performance. Overall conversion of ethylbenzene with product selectivity under various conditions is shown in Table 2.

The catalyst reusability has been found low after the second cycle (Table 2, entry 6), which shows a drop to 23.6% conversion with lower selectivity towards ACP, BZL, and PE. This may be due to the increase of average particle size up to 7.48 nm as shown in Figure 6. Here only 0.3 wt% drop of gold loading was observed (Table 1, entry 5). The similar findings of lower yield have been reported by Martin et al. (2017) on Au/ZnO for cyclohexane even after increasing the reaction time to 32 h [ 43].

### 3.5. Effect of solvent, oxidant molar ratio, catalyst weight, Au wt%, and volume

**Figure 13:**
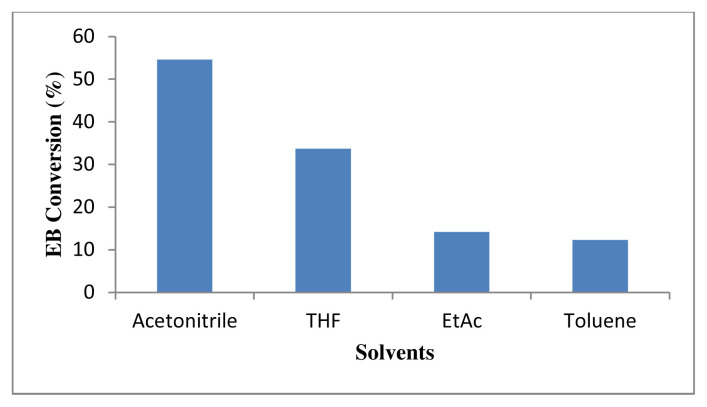
Effect of type of solvents to conversion of 0.2 M ethylbenzene oxidation using 50 mg of 4 wt% Au/ZnO at 100 °C for 24 h with 1 mmol TBHP.

To optimize the reaction conditions, the selection of a suitable solvent was also studied. Figure 13 shows the effect of solvent towards the oxidation of ethylbenzene in the presence of 4 wt% Au/ZnO catalyst in reaction temperature at 100 °C for 24 h. The solvents tested in this study were acetonitrile, tetrahydrofuran (THF), ethyl acetate, and toluene. Acetonitrile shows the highest conversion of 54.6%, whereas the lowest conversion of 33.7% was observed for THF. Toluene shows the lowest conversion, while ethyl acetate fared slightly better. The decrease in the catalytic activity of Au/ZnO in different solvents are in the order of acetonitrile > THF > ethyl acetate > toluene. This could be possibly due to the unfavourable polarity or dielectric constant towards the intermediate as proposed by Biradar et al. [13], and possibly due to the larger solvent molecules that block the catalytic sites [58]. Generally, dipolar aprotic solvents such as THF gave better conversion compared to the nonpolar solvents such as toluene and xylene, which have lower dipole moments and dielectric constants. Solvents with higher dielectric constants promote bond dissociation, while solvents with lower dielectric constants neutralise the charged species by forming bonds [59]. In addition, solvents with lower dipole moments and dielectric constants are hydrophobic and are detrimental to the conversion. Habibi et al. reported that the conversion reaction was inhibited in water, as the lone pair of electrons of the oxygen atom in the water molecules tends to block the active site of Au [20, 60 ]. Polar solvents are also favourable for the oxidation reaction such as the oxidation of cyclohexane, where the conversion increases with the solvent polarity. Jing et al. suggest that the π-bonds in the polar solvent reacts with the transition metal ions to form an intermediate, which promotes the reaction [ 61]. The type of solvent is relatively important, since some solvents are cooxidants; in addition, they produce another oxidizing agent, which increases the rate of conversion [62]. Acetonitrile is known to produce peroxy carboximidic acid, a powerful oxidizing agent, while THF promotes a radical oxidation process; thus, these solvents gave higher conversion of ethylbenzene [62]. Hence, it is concluded that ethylbenzene conversion is optimized in acetonitrile as the solvent, regardless of the higher selectivity of other solvents.

**Figure 14:**
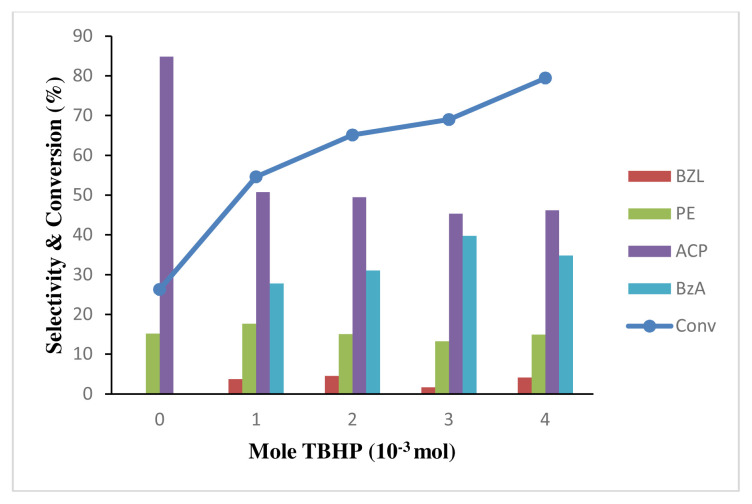
Effect of oxidant to substrate ratio for conversion of 0.2 M ethylbenzene oxidation and product selectivity using 50 mg of 4 wt% Au/ZnO at 100 °C for 24 h.

Figure 14 shows the oxidation in the absence of TBHP (*tert*-butyl hydroxyl peroxide) at 26.3% conversion of EB with high selectivity towards acetophenone at 84.8% and significant selectivity of PE at 15.2%. The reaction without TBHP was still taking place as oxygen gas from ambient pressure was involved in the reaction. It is noted from Figure 14 that no benzaldehyde was formed, since the reaction without oxidation agent favors the formation of acetophenone and 1-phenyethanol only, due to the limitation of oxygen from surrounding atmosphere. Therefore, no benzoic acid was formed as benzaldehyde. The increase in TBHP concentration gave a linear increase in the EB conversion, as more TBHP radicals that supplies oxygen were available for the reaction. The selectivity of ACP and PE are almost constant from 2 to 4 mole of TBHP. Addition of activated oxygen of cocoordinated TBHP into a C–H bond of the methylene group in EB produced PE. Subsequent abstraction of alcoholic OH hydrogen and the CH hydrogen of 1-phenylethanol would yield ACP or BZL. Both of the product can be further oxidize to benzoic acid [63]. Increasing of TBHP allow more secondary oxidation of other byproducts to occur, which can be observed where ACP and PE remain almost stagnant while producing more benzoic acid from further oxidation of benzaldehyde, which appear to reduce as benzoic acid increase [64]. Small amount of benzaldehyde is observed with addition of TBHP because benzaldehyde went autoxidation to form benzoic acid with the presence of oxidation agent [65]. The catalyst also appear to be effective at lower oxidant to ethylbenzene molar ratio (1:1) and was able to achieve more than 50% conversion of EB compared to other reported oxidation of EB that uses higher oxidant to substrate molar ratio [64, 66 ]

Other peroxide agents such as H_2_O_2_ (37% of H_2_O_2_) used with no catalytic activity and selectivity were observed. This is possibly due to the significant amount of water in H_2_O_2_, as water deactivates the active sites of the catalyst [60]. Since TBHP was the source of oxygen for the reaction, it is suggested that the rate of ethylbenzene oxidation is proportional to the concentration of TBHP.

**Figure 15:**
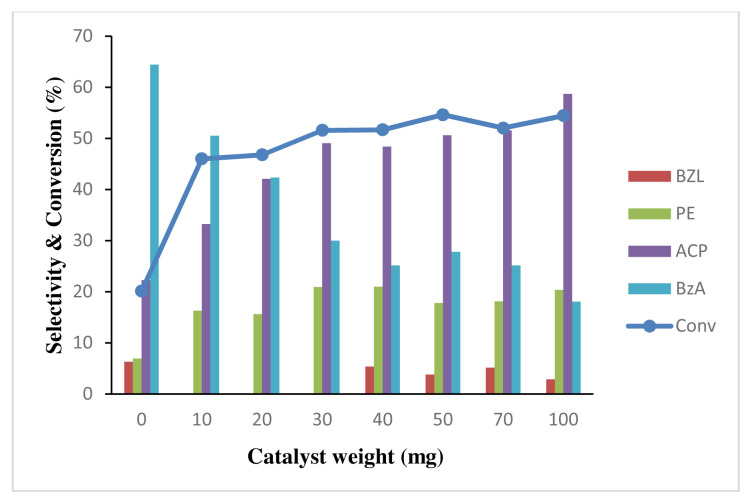
Effect of catalyst weight to conversion of 0.2 M ethylbenzene oxidation and product selectivity using 4 wt% Au/ZnO at 100 °C for 24 h with 1 mmol of TBHP.

To study the effect of catalyst weight on the oxidation reaction, TBHP and EB at 1:1 ratio was kept constant. As the catalyst was present at lower weight (10 mg), the conversion of EB increased to 46% as shown in Figure 15. The conversion gradually increased from 10 mg to 100 mg, suggesting that the amount becomes rate limiting at 10 mg onwards. As the catalyst amount exceeded 50 mg, the EB conversion slightly drops and remains unchanged probably due to the diffusion control factor [67]. Selectivity for ACP increases as catalyst weight increases, assuming that the presence of gold nanoparticles favors the reaction pathway toward ACP. It is also observed the highest selectivity of benzoic acid (64%) in Figure 15, which shows the reaction without catalyst. It is possibly due to the presence of TBHP, most benzaldehyde is further oxidized to benzoic acid. However, the selectivity of benzoic acid is reduced from 10 mg to 40 mg catalyst possibly because most oxygen molecules from TBHP were adsorbed on the catalyst surface as depicted in the proposed reaction mechanism in Scheme. The surface area for the catalyst was increased, as more catalyst was used in the reaction. Therefore, more TBHP was adsorbed on the catalyst surface. The selectivity benzoic acid is constant around 25% from 40 mg to 100 mg due to lack of oxygen radical to oxidize benzaldehyde.

**Figure 16:**
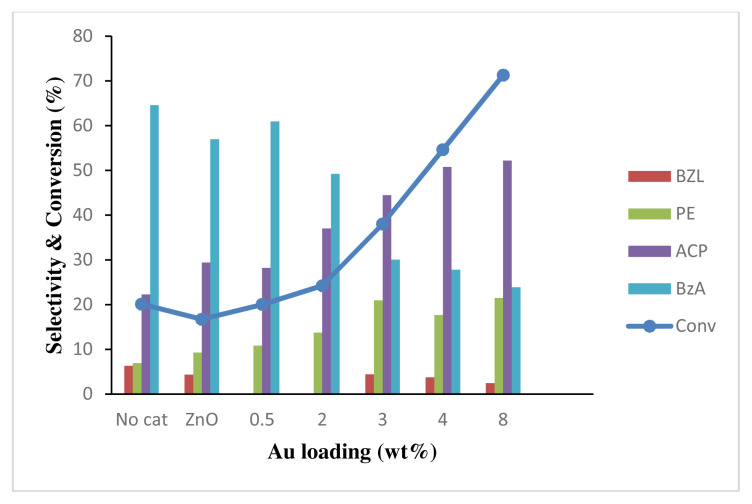
Effect of gold loading to conversion of 0.2 M ethylbenzene oxidation and product selectivity using 50 mg of Au/ZnO at 100 °C for 24 h with 1 mmol of TBHP.

The rate of EB oxidation also depends on the amount of gold loading (wt%) in the catalyst, as shown in Figure 16. In the absence of the catalyst, the oxidation reaction shows 20.1% conversion of EB. ZnO shows slight reduction in EB oxidation conversion, as it is likely that ZnO adsorbed TBHP during reaction and significantly inhibited TBHP from reacting with ethylbenzene. Similar conversion for no catalyst was observed when 0.5 wt% Au on ZnO was used as catalyst with almost similar selectivity with ZnO catalyst. The product selectivity towards BzA was decreased, while the selectivity towards ACP increased, as more Au metal loading was used. The increasing gold loadings demonstrated higher conversion due to the presence of more Au nanoparticles that provide additional active sites for the catalytic activity. Therefore, the conversion of EB is proportional to the amount of gold loading, as more active sites are available. However, higher metal nanoparticles loading also increases the tendency towards particle agglomeration and reduces the catalytic performance [ 67]. Some studies reported that higher conversion could be achieved with larger size of gold nanoparticles between 6.9 and 8.4 nm [13, 68 ]. Surprisingly, Au/ZnO at high gold loading provides high conversion. Further study may be required to determine the maximum gold loading efficiency of the catalyst. In term of selectivity, the benzoic acid selectivity using ZnO support is reduced to 54.7% due to adsorption of TBHP. The selectivity of benzoic acid is reduced significantly from 0.5 to 8 wt% Au as more TBHP was adsorbed on the active sites of Au metal catalyst. The increasing Au loading had increased the active sites of the catalyst that lead to high adsorption of oxygen radicals from TBHP. Therefore, it shows less oxygen is needed for the oxidation of benzaldehyde to benzoic acid.

**Figure 17:**
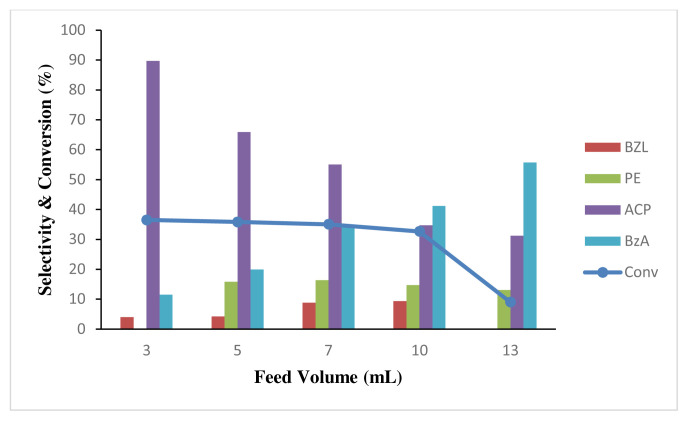
Effect of total feed volume to conversion of ethylbenzene oxidation 0.2 M and product selectivity using 50 mg of 4 wt% Au/ZnO at 100 °C for 24 h.

Volume of feed also played an important role in the catalyst performance, as shown in Figure 17, as ACN used is higher when higher feed volume was used. Five different volumes 3, 5, 7, 10, and 13 mL were tested, and the conversion percentages were determined after 24 h of reaction. As observed in Figure 17, the increase in substrate volume, which corresponds to the increase in solvent volume, led to a significant decrease in the catalytic conversion with time, similar to the results reported by Titinchi et al. [ 69]. The reaction performed at higher volume shows lower catalytic activity. The conversion of ethyl benzene and selectivity of acetophenone is disproportional with the feed volume. This could be due to the inhibition of active sites by the solvent molecules, thus creating a diffusion competition between the solvent and the substrate [58]. It is demonstrated that larger solvent volume contributes to lower conversion, as the higher solvent ratio suppresses the analyte, thereby lowering the reaction rates [69]. This finding is also supported by the results of Arshadi et al., where the catalytic activity was higher in solvent free condition than in the presence of ACN [70]. However, due to the absence of a highly efficient reflux system, a detailed analysis of the solvent-free method was not possible, as the products were completely dried out before it could be analysed using GC. Due to the same reason, almost 3 mL of volume had been lost after reaction, thus making 5 mL was the optimum volume as the volume recovered from a 3 mL volume substrate was inconsistent and sometimes insufficient for further testing using GC. It is also noted in Figure 17 that benzoic acid selectivity increases with the increasing volume of acetonitrile. This trend was expected as acetonitrile is also an oxidizing agent that can oxidize benzaldehyde to benzoic acid during reaction. Therefore, feed volume around 5 mL was sufficient to ensure acceptable ethyl benzene conversion with high acetophenone and minimum benzoic acid selectivity.

A few literature exists on gold nanoparticle based catalysts [8,12, 43, 44 ]. However, to the best of our knowledge, the use of Au/ZnO on ethylbenzene oxidation is nonexistent. Our work proves that using deposition precipitation by urea is able to produce high loading of gold nanoparticles with small ranging from 1 to 5 nm. The comprehensive catalytic study has also been performed on the other products such as 1-phenylethanol, benzaldehyde, acetophenone, and benzoic acid with lower oxidant to ethylbenzene ratio as compared with other catalysts [ 13, 64, 66 ].

## 4. Discussion

The deposition precipitation by urea approach was used to successfully produce small gold nanoparticles on zinc oxide supports, and the catalyst was demonstrated to be a promising and efficient catalyst for the oxidation of ethylbenzene. For the catalytic oxidation of ethylbenzene, the effect of using very small nanosized gold (3 nm) on ZnO demonstrated high selectivity towards acetophenone as the major product, while a moderate selectivity towards other products such as 1-phenylethanol, benzaldehyde, and benzoic acid were observed. Since benzaldehyde underwent autooxidation to generate benzoic acid in the presence of TBHP oxidation agent, a little amount of benzaldehyde was detected. Gold loading, calcination treatment, solvent type, low oxidant molar ratio, and low solvent volume were found to have a definite impact on ethylbenzene conversion.

## Figures and Tables

**Figure 10:**
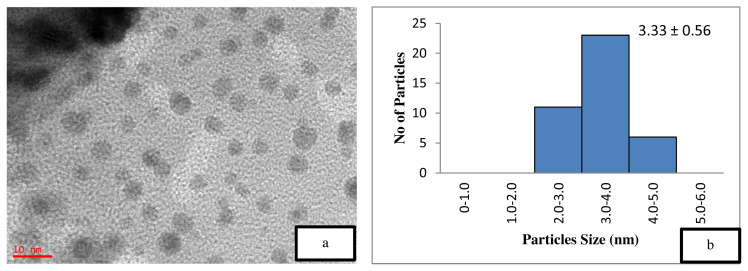
TEM image of (a) calcined 8.0 wt% Au/ZnO at 10 nm and (b) particle distribution graph.

**Scheme:**
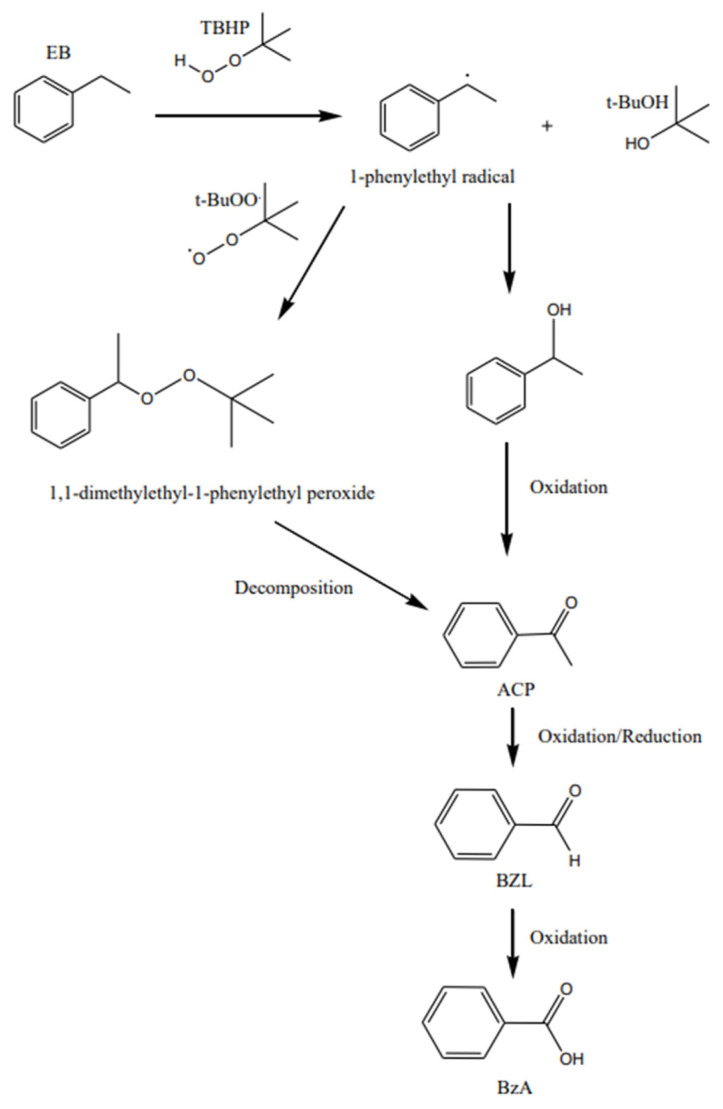
Proposed reaction mechanism for ethylbenzene oxidation with gold nanoparticles on zinc oxide and TBHP as the oxidant under reflux.

**Table 1:** XRF gold loading, BET surface area, pore size, pore volume, and HRTEM average gold particle size.

Entry	Catalyst	Au Loading (wt%)	BET surface area (m^2^/g)	Pore Volume (m^3^/g)	Pore size (Å)	Particles Size (nm)
1	ZnO (uncalcined)	0	22.32	0.031	55.38	-
2	ZnO (calcined)	0	10.57	0.035	132.72	-
3	4 wt % Au/ZnO (uncalcined)	3.7	26.69	0.032	48.56	1.71 ± 0.33
4	4 wt % Au/ZnO (calcined)	3.4	16.20	0.073	179.98	2.85 ± 0.62
5	4 wt % Au/ZnO (spent)	3.1	19.49	0.058	120.64	7.48 ± 1.93
6	0.5 wt % Au/ZnO (calcined)	0.4	12.78	0.064	201.36	1.59 ± 0.25
7	2.0 wt % Au/ZnO (calcined)	1.9	14.54	0.069	191.24	2.31 ± 0.42
8	8.0 wt % Au/ZnO (calcined)	7.1	18.39	0.089	194.63	3.33 ± 0.56

**Table 2:** Conversion of ethylbenzene with product selectivity under various conditions.

Entry	Catalyst	EB Conversion (%)	Selectivity (%)			
			BZL	PE	ACP	BzA
1	No catalyst	20.1	6.3	6.9	22.3	64.6
2	ZnO (a)	20.6	0	5.8	18.4	77.2
3	ZnO (b)	16.7	4.4	9.3	29.4	57.0
4	4 wt% Au/ZnO (a)	40.0	8.0	11.7	27.0	53.5
5	4 wt% Au/ZnO (b)	54.6	3.8	17.7	50.8	27.8
6	4 wt% Au/ZnO (c)	23.6	0	5.6	22.8	71.6
7	4 wt% Au/ZnO (d)	26.3	0	15.2	84.8	0
8	0.5 wt% Au/ZnO (b)	20.0	0	10.8	28.2	60.9
9	2.0 wt% Au/ZnO (b)	24.4	0	13.7	37.0	49.2
10	2.0 wt% Au/ZnO (e)	21.8	0	13.6	35.3	51.2
11	3.0 wt% Au/ZnO (b)	38.0	4.4	21.1	44.5	30.0
12	8.0 wt % Au/ZnO (b)	71.3	2.5	21.5	52.2	23.9

(a) Uncalcined catalyst, (b) calcined catalyst, (c) spent/recycled catalyst, (d) catalyst without tBHP, (e) catalyst stored up to 8 months in refrigerator.

## References

[1] Philip A Lihavainen J Keinänen M Pakkanen TT Gold nanoparticle-decorated halloysite nanotubes-selective catalysts for benzyl alcohol oxidation Applied Clay Science 2017 143 80 88

[2] Torres CC Alderete JB Pecchi G Campos CH Reyes P Heterogeneous hydrogenation of nitroaromatic compounds on gold catalysts influence of titanium substitution in MCM-41 mesoporous supports Applied Catalysis A General 2016 517 110 119

[3] Fang X Sharma M Stennett C Gill PP Optical sensitisation of energetic crystals with gold nanoparticles for laser ignition Combustion and Flame 2017 183 15 21

[4] Jiménez-Pérez ZE Singh P Kim YJ Mathiyalagan R Kim DH Applications of panax ginseng leaves-mediated gold nanoparticles in cosmetics relation to antioxidant, moisture retention, and whitening effect on B16BL6 cells Journal of Ginseng Research 2017 42 3 327 333 29983614 10.1016/j.jgr.2017.04.003 PMC6026357

[5] Khan S Runguo W Tahir K Jichuan Z Zhang L Catalytic reduction of 4-nitrophenol and photo inhibition of pseudomonas aeruginosa using gold nanoparticles as photocatalyst Journal of Photochemistry and Photobiology Biology 2017 170 181 187 10.1016/j.jphotobiol.2017.04.006 28437746

[6] Tan KH Lee HW Chen JW Dee CF Majlis BY Self-assembled heteroepitaxial AuNPs/SrTiO3 influence of AuNPs Size on SrTiO3 band gap tuning for visible light-driven photocatalyst The Journal of Physical Chemistry C 2017 121 25 13487 13495

[7] Pflästerer D Hashmi ASK Gold catalysis in total synthesis–recent achievements Chemical Society Reviews 2016 45 5 1331 1367 26673389 10.1039/c5cs00721f

[8] Fujita T Horikawa M Takei T Murayama T Haruta M Correlation between catalytic activity of supported gold catalysts for carbon monoxide oxidation and metal–oxygen binding energy of the support metal oxides Chinese Journal of Catalysis 2016 37 10 1651 1655

[9] Udawatte N Lee M Kim J Lee D Well-defined Au/ZnO nanoparticle composites exhibiting enhanced photocatalytic activities ACS Applied Materials & Interfaces 2011 3 11 4531 4538 22029573 10.1021/am201221x

[10] Wu H Wang L Zhang J Shen Z Zhao J Catalytic oxidation of benzene, toluene and p-xylene over colloidal gold supported on zinc oxide catalyst Catalysis Communications 2011 12 10 859 865

[11] Li W Du L Jia C Si R Support effect of zinc tin oxide on gold catalyst for CO oxidation reaction Chinese Journal of Catalysis 2016 37 10 1702 1711

[12] Yazid H Adnan R Hamid SA Farrukh MA Synthesis and characterization of gold nanoparticles supported on zinc oxide via the deposition-precipitation method Turkish Journal of Chemistry 2010 34 4 639 650

[13] Biradar AV Asefa T Nanosized gold-catalyzed selective oxidation of alkyl-substituted benzenes and n-alkanes Applied Catalysis A General 2012 435–436 19 26

[14] Lin X Zhao S Chen Y Fu L Zhu R Nitrogen-doped carbon cobalt grafted on graphitic carbon nitride catalysts with enhanced catalytic performance for ethylbenzene oxidation Journal of Molecular Catalysis A Chemical 2016 420 11 17

[15] Wilson K Book review of green chemistry and catalysis Applied Organometallic Chemistry 2007 21 1002

[16] Dapurkar SE Shervani Z Yokoyama T Ikushima Y Kawanami H Supported gold nanoparticles catalysts for solvent-free selective oxidation of benzylic compounds into ketones at 1 atm O2 Catalysis Letters 2009 130 1–2 42 47

[17] Zhu M Wei X Li B Yuan Y Copper-triethanolamine complex as efficient and active catalyst for selective oxidation of alkylarenes to phenyl ketones by tert-butylhydroperoxide Tetrahedron Letters 2007 48 52 9108 9111

[18] Hossain MAM Ali ME Abd Hamid SB Rahman MM Catalytic Oxidation of Alkyl Benzene Advanced Materials Research 2015 1109 248 252

[19] Qiao B Liang JX Wang A Liu J Zhang T Single atom gold catalysts for low-temperature CO oxidation Chinese Journal of Catalysis 2016 37 10 1580 1586

[20] Habibi D Faraji AR Arshadi M Fierro JLG Characterization and catalytic activity of a novel Fe nano-catalyst as efficient heterogeneous catalyst for selective oxidation of ethylbenzene, cyclohexene, and benzylalcohol Journal of Molecular Catalysis A Chemical 2013 372 90 99

[21] Vetrivel S Pandurangan A Side-chain oxidation of ethylbenzene with tert-butylhydroperoxide over mesoporous Mn-MCM-41 molecular sieves Journal of Molecular Catalysis A Chemical 2004 217 1 165 174

[22] Zhang G Wang D Feng P Shi S Wang C Synthesis of zeolite beta containing ultra-small CoO particles for ethylbenzene oxidation Chinese Journal of Catalysis 2017 38 7 1207 1215

[23] Oliveira APS Gomes IS Neto ASB Oliveira AC Filho JM Catalytic performance of MnFeSi composite in selective oxidation of styrene, ethylbenzene and benzyl alcohol Molecular Catalysis 2017 436 29 42

[24] Besson M Gallezot P Selective oxidation of alcohols and aldehydes on metal catalysts Catalysis Today 2000 57 1 127 141

[25] Kluytmans JHJ Markusse AP Kuster BFM Marin G Schouten JC Engineering aspects of the aqueous noble metal catalysed alcohol oxidation Catalysis Today 2000 57 1–2 143 155

[26] Haruta M Gold as a novel catalyst in the 21st century: Preparation, working mechanism and applications Gold bulletin 2004 37 1 27 36

[27] Perego C Villa P Catalyst preparation methods Catalysis Today 1997 34 3–4 281 305

[28] Akhtar MJ Ahamed M Kumar S Khan MM Ahmad J Zinc oxide nanoparticles selectively induce apoptosis in human cancer cells through reactive oxygen species International Journal of Nanomedicine 2012 7 845 22393286 10.2147/IJN.S29129 PMC3289443

[29] Perumal V Hashim U Gopinath SC Haarindraprasad R Poopalan P A new nano-worm structure from gold-nanoparticle mediated random curving of zinc oxide nanorods Biosens Bioelectron 2016 78 14 22 26584078 10.1016/j.bios.2015.10.083

[30] Chatterjee AK X-ray diffraction Ramachandran VS Beau JJ Handbook of analytical in concrete science and technology Norwich, NY, USA William Andrew Publishing 2001 275 332

[31] Ndolomingo MJ Meijboom R Selective liquid phase oxidation of benzyl alcohol to benzaldehyde by tert-butyl hydroperoxide over γ-Al2O3 supported copper and gold nanoparticles Applied Surface Science 2017 398 19 32

[32] Al-Hada NM Saion EB Shaari AH Kamarudin MA Flaifel MH A facile thermal-treatment route to synthesize ZnO nanosheets and effect of calcination temperature PloS one 2014 9 8 e103134 25093752 10.1371/journal.pone.0103134 PMC4122363

[33] Stacey P Kauffer E Moulut JC Dion C Beauparlant M An international comparison of the crystallinity of calibration materials for the analysis of respirable-quartz using x-ray diffraction and a comparison with results from the infrared KBr disc method The Annals of Occupational Hygiene 2009 53 6 639 649 19531809 10.1093/annhyg/mep038

[34] Kołodziejczak-Radzimska A Markiewicz E Jesionowski T Structural characterisation of ZnO particles obtained by the emulsion precipitation method Journal of Nanomaterials 2012 2012 1 9

[35] Ismail AA Harraz FA Faisal M El-Toni AM Al-Hajry A A sensitive and selective amperometric hydrazine sensor based on mesoporous Au/ZnO nanocomposites Materials & Design 2016 109 530 538

[36] Gangopadhyay D Singh DS Sharma P Mishra H Unnikrishnan VK Spectroscopic and structural study of the newly synthesized heteroligand complex of copper with creatinine and urea Spectrochimica acta part 4 Molecular and Biomolecular Spectroscopy 2016 154 200 206 10.1016/j.saa.2015.10.028 26529636

[37] Zanella R Louis C Influence of the conditions of thermal treatments and of storage on the size of the gold particles in Au/TiO2 samples Catalysis Today 2005 107–108 Supplement C 768 777

[38] Haruta M Ueda A Tsubota S Torres Sanchez RM Low-temperature catalytic combustion of methanol and its decomposed derivatives over supported gold catalysts Catalysis Today 1996 29 1 443 447

[39] Zanella R Giorgio S Henry CR Louis C Alternative methods for the preparation of gold nanoparticles supported on TiO2 The Journal of Physical Chemistry B 2002 106 31 7634 7642

[40] Zanella R Delannoy L Louis C Mechanism of deposition of gold precursors onto TiO2 during the preparation by cation adsorption and deposition–precipitation with NaOH and urea Applied Catalysis A General 2005 291 1–2 62 72

[41] Tsubota S Cunningham D Bando Y Haruta M Preparation of nanometer gold strongly interacted with TiO2 and the structure sensitivity in low-temperature oxidation of CO Studies in Surface Science and Catalysis 1995 91 227 235

[42] Dimitratos N Villa A Bianchi CL Prati L Makkee M Gold on titania effect of preparation method in the liquid phase oxidation Applied Catalysis A General 2006 311 185 192

[43] Dias Ribeiro de Sousa Martins LM Carabineiro SAC Wang J Rocha BGM Maldonado-Hodar FJ Supported Gold Nanoparticles as Reusable Catalysts for Oxidation Reactions of Industrial Significance ChemCatChem 2017 9 7 1211 1221

[44] Vourros A Garagounis I Kyriakou V Carabineiro SAC Maldonado-Hódar FJ Carbon dioxide hydrogenation over supported Au nanoparticles effect of the support Journal of CO2 Utilization 2017 19 247 256

[45] Alabbad S Adil SF Assal ME Khan M Alwarthan A Gold & silver nanoparticles supported on manganese oxide synthesis, characterization and catalytic studies for selective oxidation of benzyl alcohol Arabian Journal of Chemistry 2014 7 6 1192 1198

[46] Perkas N Zhong Z Grinblat J Gedanken Deposition of gold particles on mesoporous catalyst supports by sonochemical method, and their catalytic performance for CO oxidation Catalysis Letters 2008 120 19 24

[47] Thommes M Kaneko K Neimark AV Olivier JP Rodriguez-Reinoso F Physisorption of gases, with special reference to the evaluation of surface area and pore size distribution (IUPAC Technical Report) Pure and Applied Chemistry 2015 87 9–10 1051 1069

[48] Wang D Hao Z Cheng D Shi X Influence of the calcination temperature on the Au/FeOx/Al2O3 catalyst Journal of Chemical Technology and Biotechnology 2006 81 7 1246 1251

[49] Yesmurzayeva NN Nurakhmetova ZA Tatykhanova GS Selenova BS Kudaibergenov SE Catalytic Activity of Gold and Silver Nanoparticles Supported on Zinc Oxide Supramolecular Catalysis 2015 2 1

[50] Carabineiro SAC Supported Gold Nanoparticles as Catalysts for the Oxidation of Alcohols and Alkanes Frontiers in Chemistry 2019 7 702 10.3389/fchem.2019.00702 PMC6848162 31750289

[51] Tang Y Xu J Wang F Zheng Y Zhang Z Mechanism study on the oxidation of ethylbenzene a theoretical and computational approach Computational and Theoretical Chemistry 2020 1188 112974

[52] Richardson WH Thomson SA Search for electron-transfer decomposition and the production of electronically excited state species in the thermolysis of p-(dimethylamino)phenyl-substituted dialkyl peroxides The Journal of Organic Chemistry 1982 47 23 4515 4520

[53] Du M Sun D Yang H Huang J Jing X Odoom-Wubah T Influence of Au particle size on Au/TiO2 catalysts for CO oxidation The Journal of Physical Chemistry C 2014 118 33 19150 19157

[54] Riaz S Bashir M Raza MA Mahmood A Naseem S Effect of Calcination on Structural and Magnetic Properties of Co-Doped ZnO Nanostructures IEEE Transactions on Magnetics 2015 51 11 1 4 26203196

[55] Jain A Zhao X Kjergaard S Stagg-Williams SM Effect of aging time and calcination on the preferential oxidation of CO over Au supported on doped ceria Catalysis letters 2005 104 3 191 197

[56] Masatake H When Gold Is Not Noble: Catalysis by Nanoparticles The Chemical Record 2003 3 2 75 87 12731078 10.1002/tcr.10053

[57] Kamat PV Photophysical, photochemical and photocatalytic aspects of metal nanoparticles The Journal of Physical Chemistry B 2002 106 32 7729 7744

[58] Bhoware SS Shylesh S Kamble KR Singh AP Cobalt-containing hexagonal mesoporous molecular sieves (Co-HMS) synthesis, characterization and catalytic activity in the oxidation reaction of ethylbenzene Journal of Molecular Catalysis A Chemical 2006 255 1 123 130

[59] Carey FA Sundberg RJ Chemical bonding and molecular structure In: Advanced organic chemistry part A structure and mechanisms Boston, MA, USA Springer 2007 1 117

[60] Arshadi M Ghiaci M Rahmanian A Ghaziaskar H Gil A Oxidation of ethylbenzene to acetophenone by a Mn catalyst supported on a modified nanosized SiO2/Al2O3 mixed-oxide in supercritical carbon dioxide Applied Catalysis B Environmental 2012 119–120 81 90

[61] Jing B Li J Qin Z Selective oxidation of cyclohexane over Co-APO-5 effects of solvent and modification method on the catalytic performance Journal of Fuel Chemistry and Technology 2016 44 10 1249 1258

[62] Andrade Z Carlos K Alves LM Environmentally benign solvents in organic synthesis current topics Current Organic Chemistry 2005 9 2 195 218

[63] Kanjina W Trakarnpruk W Mixed metal oxide catalysts for the selective oxidation of ethylbenzene to acetophenone Chinese Chemical Letters 2011 22 401 404

[64] Unnarkat AP Singh S Kalan S Ethylbenzene oxidation using cobalt oxide supported over SBA-15 and KIT-6 Materials Today Proceedings 2021 45 3991 3996

[65] Sankar M Nowicka E Carter E Murphy DM Knight DW The benzaldehyde oxidation paradox explained by the interception of peroxy radical by benzyl alcohol Nature Communications 2014 5 1 3332 10.1038/ncomms4332 24567108

[66] Chaudhary V Sharma S Study of ethylbenzene oxidation over polymer-silica hybrid supported Co (II) and Cu (II) complexes Catalysis Today 2021 375 601 613

[67] Ji D Xi N Li G Dong P Li H Hydrotalcite-based CoxNiyAl1Ox mixed oxide as a highly efficient catalyst for selective ethylbenzene oxidation Molecular Catalysis 2021 508 111579

[68] Haider P Kimmerle B Krumeich F Kleist W Grunwaldt JD Gold-catalyzed aerobic oxidation of benzyl alcohol effect of gold particle size on activity and selectivity in different solvents Catalysis Letters 2008 125 3 169 176

[69] Titinchi SJJ Von Willingh G Abbo HS Prasad R Tri and tetradentate copper complexes a comparative study on homogeneous and heterogeneous catalysis over oxidation reactions Catalysis Science Technology 2015 5 1 325 338

[70] Arshadi M Ghiaci M Highly efficient solvent free oxidation of ethylbenzene using some recyclable catalysts the role of linker in competency of manganese nanocatalysts Applied Catalysis A General 2011 399 1 75 86

